# Supplementary material for: Temporo-cerebellar connectivity underlies timing constraints in audition
Source: eLife. 2021 Sep 20;10:e67303. doi: 10.7554/eLife.67303 (PMC8480974; doi:10.7554/eLife.67303)
Supplement: Supplementary file 1. [file elife-67303-supp1.docx]

**Supplemental Information**

**Temporal cortex-cerebellum connectivity underlies timing constraints on audition**

Anika Stockert, Michael Schwartze, David Poeppel, Alfred Anwander, Sonja A. Kotz

**Supplementary File 1a. Characteristics of the studied groups**

| **Group** | **Age (mean ± SD)** | **Sex (f/m)** | **HI (mean ± SD)** | **LE (mean ± SD)** |
| --- | --- | --- | --- | --- |
| **CG** | 53.8 ± 8.9 | 5/7 | + 90.4 ± 11.0 | 10.5 ± 1.2 |
| **LG** | 53.1 ± 8.6 | 5/7 | + 90 ± 15.4 | 10.7 ± 1.3 |

Mean (±SD) age, handedness index (HI, Edinburgh Handedness Inventory), highest level of formal education in years (LE) and gender ratios (m, male; f, female) for control (CG) and lesion (LG) group.

**Supplementary File 1b. Demographic and clinical information for patients**

| **Subject** | **Age** | **Sex** | **TSL** | **HI** | **NIHSS** | **Lesion location** | **LV (mL)** |
| --- | --- | --- | --- | --- | --- | --- | --- |
| **01** | 56 | m | 150 | +100 | 4 | ITG, MTG, STS, PHG, FG, AG, OGg, LG | 144.6 |
| **02** | 59 | m | 102 | +100 | 1 | STG, SMG, AG, In | 28.1 |
| **03** | 45 | f | 87 | +60 | 1 | STG, SMG | 27.4 |
| **05** | 52 | f | 86 | +100 | 3 | STG, STS, AG, In, EC | 50.9 |
| **06** | 67 | m | 83 | +100 | 1 | STG, STS, AG | 23.5 |
| **07** | 61 | m | 70 | +90 | 3 | MTG, STG, STS | 80.1 |
| **08** | 57 | f | 35 | +60 | 1 | MTG, SMG, AG, PoG | 60.2 |
| **09** | 49 | f | 36 | +100 | 0 | MTG, ITG | 24.4 |
| **10** | 41 | m | 33 | +100 | 2 | PT, In, EC | 5.4 |
| **11** | 39 | f | 18 | +100 | 2 | PT, SMG, In, EC, PoG | 56.8 |
| **12** | 61 | m | 20 | +90 | 1 | STG, SMG, In, EC, Pu, Cau | 41.5 |
| **13** | 50 | m | 12 | +80 | 2 | PT, SMG, In, EC | 39.5 |

Abbreviations: m, male; f, female; TSL, time since lesion in months; HI, Handedness index (based on Edinburgh Handedness Inventory); lesion location: temporal lobe: ITG, inferior temporal gyrus; MTG, middle temporal gyrus; STG, superior temporal gyrus; STS, superior temporal sulcus; PT, planum temporale; PHG, parahippocampal gyrus; FG, fusiform gyrus; parietal lobe: AG, angular gyrus; SMG, supramarginal gyrus; PoG, postcentral gyrus; occipital lobe: OGg, occipital gyri; LG, lingual gyrus; subcortical: In, insula; EC, external/extreme capsule; Pu, putamen; Cau, caudate nucleus; LV, lesion volume in milliliter (mL).

**Supplementary File 1c. Language pathology**

| **Subject** | **Aphasia description** |
| --- | --- |
| **01** | Moderate fluent aphasia with word-finding difficulties, semantic paraphasias and impaired auditory comprehension (ALLOC classification: Wernicke’s aphasia) |
| **02** | Residual conduction aphasia with impaired repetition, lexical retrieval, phonematic paraphasias and mild receptive morphosyntactic deficits (ALLOC classification: no classification/residual symptoms) |
| **03** | Mild expressive phonological disorder with impaired phonological working memory and phonematic paraphasias (ALLOC classification: no classification/residual symptoms) |
| **05** | Mild expressive phonological and lexical disorder with word-finding difficulties, semantic paraphasias, impaired phonological working memory and morphosyntactic deficits (ALLOC classification: no classification/residual symptoms) |
| **06** | Mild expressive phonological disorder with word-finding difficulties and phonematic paraphasias (ALLOC classification: no classification/residual symptoms) |
| **07** | Moderate fluent aphasia with impaired repetition, phonematic and semantic paraphasias, word-finding difficulties, paragrammatism and impaired auditory comprehension for words and sentences (ALLOC classification: Wernicke’s aphasia) |
| **08** | Mild expressive phonological disorder with impaired word retrieval, phonematic paraphasias, paralexias and paragraphia (ALLOC classification: no classification/residual symptoms) |
| **09** | Exclusively impaired word retrieval (ALLOC classification: no classification/residual symptoms) |
| **10** | Mild expressive phonological disorder with phonematic paraphasias (ALLOC classification: no classification/residual symptoms) |
| **11** | Mild expressive phonological disorder with phonematic paraphasias, word-finding difficulties, dyslexia, and dysgraphia (ALLOC classification: no classification/residual symptoms) |
| **12** | Moderate expressive phonological-lexical disorder with phonematic paraphasias, word-finding difficulties, dyslexia, and dysgraphia (ALLOC classification: amnestic aphasia) |
| **13** | Mild expressive phonological disorder with phonematic paraphasias, impaired word retrieval, repetition, and lexical decisions (ALLOC classification: no classification/residual symptoms) |

ALLOC classification procedure: computer-based non-parametric discrimination analysis program that applies AAT subtest scores to classify patients with language disorders (Huber, Poeck, & Willmes, 1984).

**Supplementary File 1d. Between group comparisons of temporal order and discrimination thresholds**

| **Variable** | **Group** | | **Test statistics** |
| --- | --- | --- | --- |
| ***Task*** | ***Patients (N=12)*** | ***Controls (N=12)*** | ***Group effects*** |
| Temporal order | 282.7±264.7 | 101.2±102.1 | U = 114, p = .007 |
| Micropattern | 19.9±36.3 | 4.2±1.9 | U = 120.5, p = .002 |

Mean thresholds in milliseconds (±SD) and non-parametric test statistic for between-subject comparisons (Mann-Whitney-U test statistics, exact p-values (one-sided)) of temporal order and micropattern discrimination thresholds.

**Supplementary File 1e. Correlations between task performance and lesion volume or hearing loss (0.5-2 kHz)**

| **Variable** | **LV** | | **dBHL 0.5 kHz** | | **dBHL 1 kHz** | | **dBHL 1.5 kHz** | | **dBHL 2 kHz** | |
| --- | --- | --- | --- | --- | --- | --- | --- | --- | --- | --- |
| ***Task*** | **Test statistics** | | | | | | | | | |
| Temporal order | -.098 | .761 | -.135 | .675 | -.312 | .323 | .104 | .749 | .174 | .590 |
| Micropattern | .349 | .266 | .098 | .762 | -.008 | .980 | .013 | .967 | .185 | .565 |
| ***Feature*** | *r_s_* | ***p*** | *r_s_* | ***p*** | *r_s_* | ***p*** | *r_s_* | ***p*** | *r_s_* | ***P*** |
| Place (W) | .032 | .921 | .262 | .410 | .321 | .309 | .419 | .175 | .550 | .058 |
| Place (PW) | -.186 | .563 | .122 | .706 | .066 | .839 | .350 | .265 | .510 | .090 |
| Place (P) | -.395 | .204 | -.052 | .873 | .028 | .930 | .241 | .451 | .340 | .279 |
| Manner (W) | -.393 | .206 | .494 | .103 | .049 | .879 | .281 | .377 | .311 | .325 |
| Manner (PW) | -.285 | .369 | .182 | .571 | .073 | .823 | .510 | .090 | .550 | .064 |
| Fricatives (P) | -.393 | .206 | .494 | .103 | .049 | .879 | .281 | .377 | .311 | .325 |
| Voice (P) | -.270 | .396 | .308 | .330 | .093 | .775 | .050 | .876 | .126 | .697 |
| Place and Voice (P) | -.285 | .369 | .182 | .571 | .073 | .823 | .510 | .090 | .550 | .064 |

Non-parametric test statistics (Spearman's Rank Order Rho (*r_s_*) and p-values) for correlations between task performance (threshold levels, error rates) and lesion volume across all patients (N = 12).

**Supplementary File 1f. Between-subject comparison of deficit positive (LG^+^) and deficit negative (LG^-^) lesion group**

| **Variable** | **Group** | | **Test statistics** |
| --- | --- | --- | --- |
|  | ***LG+ (N=6)*** | ***LG- (N=6)*** | ***Group effects*** |
| Age | 53.5 ± 11.5 | 52.7 ± 5.5 | *t*(10) = -0.16, *p* = .88 |
| HI | 96.7 ± 5.2 | 83.3 ± 19.7 | *U* = 24.0, *p* = .39 |
| TSL | 51.7 ± 31.6 | 70.3 ± 51.9 | *t*(10) = 0.75, *p* = .47 |
| LV (mL) | 43.0 ± 26.2 | 54.0 ± 46.9 | *U* = 19.0, *p* = .94 |
| NIHSS | 2.0 ± 0.89 | 1.5 ± 1.38 | *t*(10) = -0.75, *p* = .47 |
| TT | 3.5 ± 6,3 | 6.5 ± 14.9 | *U* = 20.5, *p* = .70 |

Test statistics (Student's t-test, Mann-Whitney-U test, p-values) and mean (±SD) time since lesion in months (TSL), handedness index (HI), lesion volume in mL (LV), Token Test Score (TT) and National Institute of Health Stroke Scale score (NIHSS). Gender distribution was not different between LG^+^ and LG^-^ (Fisher's exact test *p* = .50, *N* = 12).

**Supplementary File 1g. Cognitive impairments**

| **Subject** | **Description of cognitive impairment (A: attention, M: memory, E: executive functions)** |
| --- | --- |
| **01** | A: impairment of divided attention and alertness to auditory stimuli; M: impaired verbal working memory, but normal non-verbal memory; E: none |
| **02** | A: impairment of divided attention, normal alertness; M: none; E: none |
| **03** | A: none; M: impaired verbal short-term and working memory, but normal non-verbal memory; E: none |
| **05*** | A: impaired alertness due to impaired temporal processing of auditory stimuli; M: impaired verbal short-term memory, but normal non-verbal memory; E: none |
| **06*** | A: impairment of divided attention and alertness to auditory and visual stimuli; M: none; E: none |
| **07*** | A: none; M: none; E: impairment of planning and self-control of actions |
| **08** | A: impairment of divided attention and alertness to auditory and visual stimuli; M: impaired verbal working-memory; E: none |
| **09** | A: none; M: none; E: none |
| **10*** | A: impaired alertness to auditory and visual stimuli; M: impaired verbal short-term and working memory, but normal non-verbal memory; E: none |
| **11*** | A: none; M: none; E: none |
| **12*** | A: none; M: none; E: impairment of reasoning, planning and inhibition |
| **13** | A: none; M: impaired verbal working memory; E: impaired interference control |

Evaluation of cognitive impairments in attention (A), memory (M) and executive functions (E) is based on the results of a comprehensive neuropsychological test battery. Based on these neuropsychological test results we can conclude that there are no differences in cognitive impairment between the two groups (deficit positive patients, LG+, are marked with an asterisk in the table below). Regarding alertness that could have impaired task performance, we found no difference between the two patient groups.
