# Supplementary material for: Temporo-cerebellar connectivity underlies timing constraints in audition
Source: eLife. 2021 Sep 20;10:e67303. doi: 10.7554/eLife.67303 (PMC8480974; doi:10.7554/eLife.67303)
Supplement: Transparent reporting form [file elife-67303-transrepform1.pdf]

Westbrook Centre
Milton Road
Cambridge CB4 1YG, UK
P 01223 855340
W elifesciences.org
T @elife

eLife's transparent reporting form

We encourage authors to provide detailed information *within their submission* to facilitate the interpretation and replication of experiments. Authors can upload supporting documentation to indicate the use of appropriate reporting guidelines for health-related research (see EQUATOR Network), life science research (see the BioSharing Information Resource), or the ARRIVE guidelines for reporting work involving animal research. Where applicable, authors should refer to any relevant reporting standards documents in this form.

If you have any questions, please consult our Journal Policies and/or contact us: editorial@elifesciences.org.

Sample-size estimation

- You should state whether an appropriate sample size was computed when the study was being designed
- You should state the statistical method of sample size computation and any required assumptions
- If no explicit power analysis was used, you should describe how you decided what sample (replicate) size (number) to use

Please outline where this information can be found within the submission (e.g., sections or figure legends), or explain why this information doesn't apply to your submission:

No explicit power analysis was used. All available patients were selected from a patient database at the Max Planck Institute for Human Cognitive and Brain Sciences, Leipzig, Germany if they met the inclusion and exclusion criteria (see below). We identified a total 12 suitable patients that met the inclusion criteria (see section *Materials and Methods – Participants*). As our sample size is relatively small for a voxel-based lesion symptom mapping analysis, we used a subtraction analysis, and confirmed our results with a non-parametrical test (Liebermeister), suitable for smaller sample sizes.

Replicates

- You should report how often each experiment was performed
- You should include a definition of biological versus technical replication
- The data obtained should be provided and sufficient information should be provided to indicate the number of independent biological and/or technical replicates
- If you encountered any outliers, you should describe how these were handled
- Criteria for exclusion/inclusion of data should be clearly stated
- High-throughput sequence data should be uploaded before submission, with a private link for reviewers provided (these are available from both GEO and ArrayExpress)

Please outline where this information can be found within the submission (e.g., sections or figure legends), or explain why this information doesn't apply to your submission:

eLife Sciences Publications, Ltd is a limited liability non-profit non-stock corporation incorporated in the State of Delaware, USA, with company number 5030732, and is registered in the UK with company number FC030576 and branch number BR015634 at the address Westbrook Centre, Milton Road, Cambridge CB4 1YG.

Replication: No replication of the experiment was performed.
Outliers: Behavioural deficits after a stroke can vary in severity, ranging from mild impairments to the inability to perform a task. As the latter case is not equivalent to an outlier, and we were interested in comparing patients with or without deficits, we assessed patients' behaviour relative to a control group. To this end, we assigned patients performing outside two SD of the healthy control group to the patient group with impaired performance (deficit positive lesion group, LG+) (*see section Methods – Lesion Mapping and Subtraction*).
Criteria for exclusion/inclusion can be found in the section *Materials and Methods – Participants*

Statistical reporting

- Statistical analysis methods should be described and justified
- Raw data should be presented in figures whenever informative to do so (typically when N per group is less than 10)
- For each experiment, you should identify the statistical tests used, exact values of N, definitions of center, methods of multiple test correction, and dispersion and precision measures (e.g., mean, median, SD, SEM, confidence intervals; and, for the major substantive results, a measure of effect size (e.g., Pearson's r, Cohen's d)
- Report exact p-values wherever possible alongside the summary statistics and 95% confidence intervals. These should be reported for all key questions and not only when the p-value is less than 0.05.

Please outline where this information can be found within the submission (e.g., sections or figure legends), or explain why this information doesn't apply to your submission:

Statistical methods are presented in the *section Data Analysis*. We describe analysis methods for both behavioural and lesion data in the paragraphs *Language and Behavioural Data and Lesion Mapping and Analysis.* In *Figure 2A and 2B*, we use Boxplots to display the behavioural data of the 12 patients and controls.
For behavioural data, mean and SD are reported in *Table 1*, statistical test variables and effect sizes (Pearson's r) are reported in the *Results section*. Exact p-values, also for test > .05 can also be found in *Table 1*.

(For large datasets, or papers with a very large number of statistical tests, you may upload a single table file with tests, Ns, etc., with reference to sections in the manuscript.)

Group allocation

- Indicate how samples were allocated into experimental groups (in the case of clinical studies, please specify allocation to treatment method); if randomization was used, please also state if restricted randomization was applied
- Indicate if masking was used during group allocation, data collection and/or data analysis

Please outline where this information can be found within the submission (e.g., sections or figure legends), or explain why this information doesn't apply to your submission:

We evaluated patients' behaviour relative to a control group. To this end, we assigned patients performing outside two SD of the healthy control groups to the group with impaired performance (deficit positive lesion group, LG+) (*see section Methods – Lesion Mapping and Subtraction*).

Additional data files ("source data")

eLife Sciences Publications, Ltd is a limited liability non-profit non-stock corporation incorporated in the State of Delaware, USA, with company number 5030732, and is registered in the UK with company number FC030576 and branch number BR015634 at the address Westbrook Centre, Milton Road, Cambridge CB4 1YG.

- We encourage you to upload relevant additional data files, such as numerical data that are represented as a graph in a figure, or as a summary table
- Where provided, these should be in the most useful format, and they can be uploaded as "Source data" files linked to a main figure or table
- Include model definition files including the full list of parameters used
- Include code used for data analysis (e.g., R, MatLab)
- Avoid stating that data files are "available upon request"

Please indicate the figures or tables for which source data files have been provided:

As previously indicated in the original submission, by German law we are not allowed to provide open access to MRI source data. The informed consent form, signed by all participants (patients and controls) at the time of data collection, clearly states that the data will only be processed and then stored in the respective data bases of the Max Planck Institute for Human Cognitive and Brain Sciences, Leipzig, Germany. Full anonymization is also not an option, as the institute's databases facilitate the re-identification of individual data for a 10-years period after publication. In addition, there is no consent from the patients or controls for the publication of individual MRI source data on a public server. Last, it is still under debate if high resolution structural brain scans can be anonymized at all. Thus, we are bound to adhere to these legal regulations.

As scientists, we do appreciate that the sharing of source data is important and would therefore always welcome discussion in relation to these source data with interested scientists. However, we can provide the (i) behavioural source data (*see Figure 2a-D; Table 1*, (ii) lesion map data of the 12 patients (the numbers of the voi files corresponds to the individual *lesion maps in Figure 1B*), and (iii) the design file (VLSM_design.val) for the lesion analysis that allowed us to group patients into LG+ and LG-groups (*see Figure 3 A, B*).

Source Data availability

Available source data, including behavioural data and lesion maps in MNI space are publicly available through the figshare repository
(DOI: 10.6084/m9.figshare.14213393).

eLife Sciences Publications, Ltd is a limited liability non-profit non-stock corporation incorporated in the State of Delaware, USA, with company number 5030732, and is registered in the UK with company number FC030576 and branch number BR015634 at the address Westbrook Centre, Milton Road, Cambridge CB4 1YG.
